# Polymethylmethacrylate distribution is associated with recompression after vertebroplasty or kyphoplasty for osteoporotic vertebral compression fractures: A retrospective study

**DOI:** 10.1371/journal.pone.0198407

**Published:** 2018-06-01

**Authors:** Yu Hou, Qi Yao, Genai Zhang, Lixiang Ding, Hui Huang

**Affiliations:** Department of Orthopedics, Beijing Shijitan Hospital, Capital Medical University, Beijing, China; Medical College of Wisconsin, UNITED STATES

## Abstract

**Background:**

Osteoporotic vertebral compression fracture, always accompanied with pain and height loss of vertebral body, has a significant negative impact on life quality of patients. Vertebroplasty or kyphoplasty is minimal invasive techniques to reconstruct the vertebral height and prevent further collapse of the fractured vertebrae by injecting polymethylmethacrylate into vertebral body. However, recompression of polymethylmethacrylate augmented vertebrae with significant vertebral height loss and aggressive local kyphotic was observed frequently after VP or KP. The purpose of this study was to investigate the effect of polymethylmethacrylate distribution on recompression of the vertebral body after vertebroplasty or kyphoplasty surgery for osteoporotic vertebral compression fracture.

**Methods:**

A total of 281 patients who were diagnosed with vertebral compression fracture (T5-L5) from June 2014 to June 2016 and underwent vertebroplasty or kyphoplasty by polymethylmethacrylate were retrospectively analyzed. The X-ray films at 1 day and 12 months after surgery were compared to evaluate the recompression of operated vertebral body. Patients were divided into those without recompression (non-recompression group) and those with recompression (recompression group). Polymethylmethacrylate distribution pattern, including location and relationship to endplates, was compared between the two groups by lateral X-ray film. Multivariate logistic regression analysis was performed to assess the potential risk factors associated with polymethylmethacrylate distribution for recompression.

**Results:**

One hundred and six (37.7%) patients experienced recompression after surgery during the follow-up period. The polymethylmethacrylate distributed in the middle of vertebral body showed significant differences between two groups. In non-recompression group, the polymethylmethacrylate in the middle portion of vertebral body were closer to endplates than that in the recompression group (upper: t = 31.41, *p*<0.001; lower: t = 12.19, *p*<0.001). The higher percentage of the height of polymethylmethacrylate in the middle portion of vertebral body indicates the lower risk of recompression (odds ratio [OR]<0.01, *p*<0.001). The recompression group and non-recompression group showed significant difference in “contacted” polymethylmethacrylate distribution pattern (polymethylmethacrylate contacted to the both upper/lower endplates) (*χ*^2^ = 66.23, *p*<0.001). The vertebra with a “contacted” polymethylmethacrylate distribution pattern has lower risk of recompression (OR = 0.09, *p*<0.001).

**Conclusions:**

Either more polymethylmethacrylate in the middle portion of vertebral body or “contacted” polymethylmethacrylate distribution pattern had a significantly less incidence of recompression. The findings indicated that the control of polymethylmethacrylate distribution during surgery may reduce the risks of recompression after vertebroplasty or kyphoplasty.

## Introduction

Osteoporotic vertebral compression fracture (OVCF), always accompanied with pain and height loss of vertebral body, has a significant negative impact on life quality of patients. Vertebroplasty or kyphoplasty (VP or KP) is minimal invasive techniques to reconstruct the vertebral height and prevent further collapse of the fractured vertebrae by injecting polymethylmethacrylate (PMMA) into vertebral body [1]. However, recompression of PMMA augmented vertebrae with significant vertebral height loss and aggressive local kyphotic was observed frequently after VP or KP [2,3]. Evidence showed that PMMA distribution pattern as a risk factor may correlated with recompression [2,4]. Kim studied the relationship between the amount of PMMA and recompression[5]. His findings suggested that as much PMMA as possible should be used to prevent recompression [5]. However, too much PMMA would cause leakage that may lead to serious complication. The ideal operation is to use as little as possible PMMA to prevent recompression [6]. So, the distribution of PMMA may play a decisive role to prevent recompression.

The location of PMMA in vertebral body was a critical element of PMMA distribution. Molly divided vertebral body into lateral and central parts, and he found there was no significant difference between the two different PMMA distribution patterns on recompression [7]. Vertebral bodies in which PMMA is placed laterally do not appear to be at risk for collapse on the un-augmented side [7]. Zhang divided vertebral body into upper, lower and middle parts and he found that patients with PMMA distributed around both the upper and lower endplates had a lower risk of recompression when compared to patients with PMMA distributed in the middle of vertebral body [4]. Although many research divided vertebral body into anterior, middle and posterior parts to perform clinical or biomechanical analysis [8–10], the relationship between PMMA distribution by this pattern and recompression is still vague.

The distance of PMMA to the endplates was another critical element of PMMA distribution. Kim found there are non-cemented bony areas between the PMMA mass and the two endplates, which result in greater height loss in KP [2]. It is also found that the distance of PMMA to the endplates is gradually decreased under repetitive loading conditions [11]. There was a special PMMA distribution pattern which distance of PMMA to the endplates equaled to zero called “contacted”. There was no non-cemented bony area between the PMMA mass and endplates in “contacted” PMMA distribution pattern. However, the relationship of recompression and “contacted” PMMA distribution pattern was still not clear.

In this study, we retrospectively analyzed the effect of polymethylmethacrylate distribution pattern on recompression of the vertebral body after vertebroplasty or kyphoplasty surgery for osteoporotic vertebral compression fracture. We hypothesized that ideal polymethylmethacrylate distribution may reduce the risks of recompression after surgery.

## Methods

### Patients

**Fig 1:**
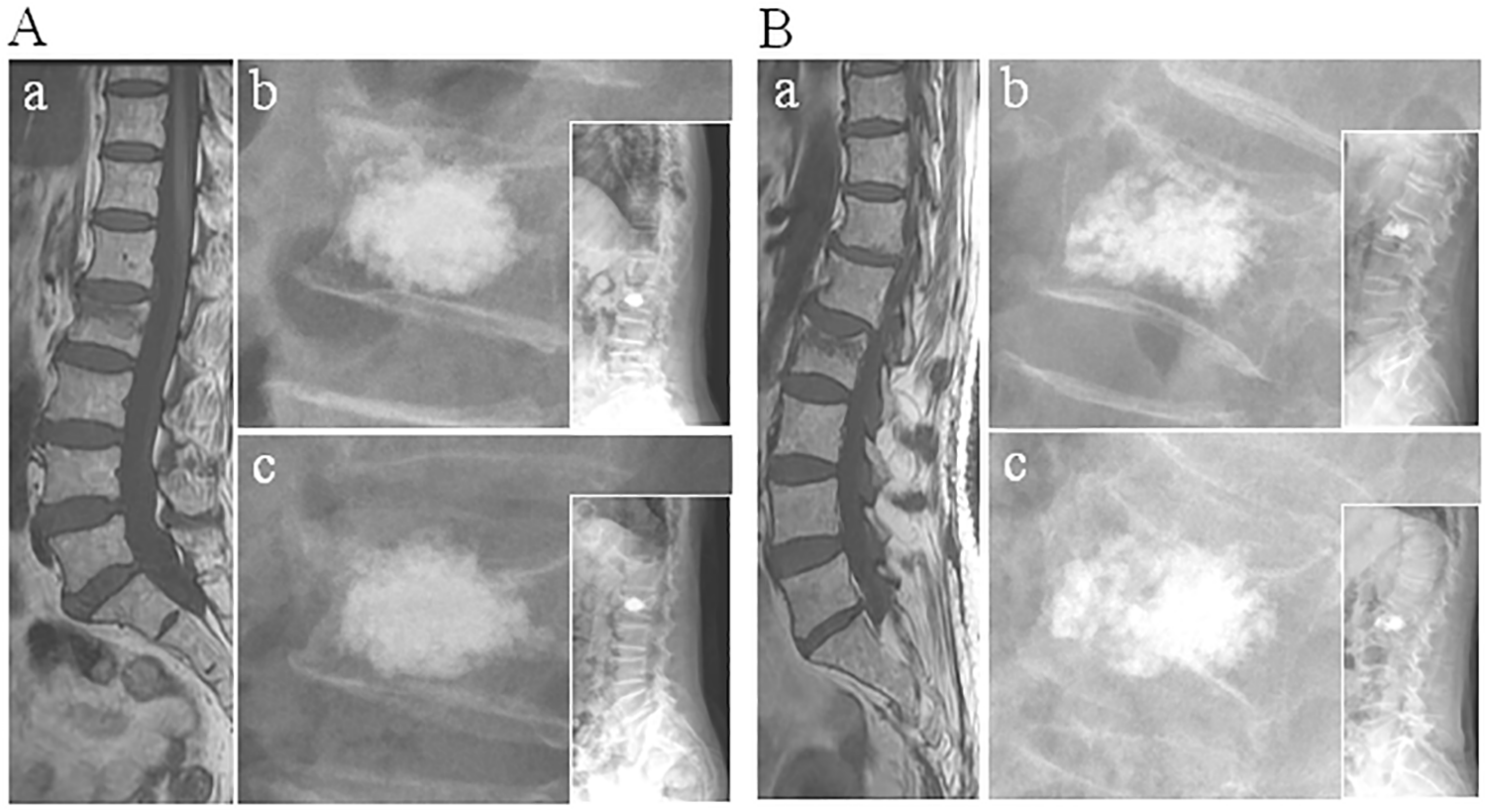
Representative MR and X-ray images of recompression and non-recompression. A: Recompression: a: MRI showed the fracture of L2. b: X-ray images taken 1 day after surgery. c: X-ray images taken 12 months after surgery. B: Non-recompression. a: MRI showed the fracture of L2. b: X-ray images taken 1 day after surgery. c: X-ray images taken 12 months after surgery. MRI = magnetic resonance imaging.

Beijing Shijitan hospital institutional review board approved the study protocol, the approval number is 201703. A total of 281 patients who were diagnosed with OVCF (T5-L5) from June 2014 to June 2016 and underwent VP or KP treatment by PMMA in our hospital were retrospectively analyzed. The painful vertebrae were confirmed based on clinical and magnetic resonance image (Fig 1). The patients were recruited whether they underwent VP/ KP by unilateral or bilateral approaches. Age, gender, bone mineral density (BMD) T-score before surgery and 12 months after surgery were also evaluated, Patients met the following criteria were excluded: 1) OVCFs caused by tumor; 2) patients with hyperparathyroidism, hyperthyroidism, pituitary adenoma, hypercortisolism or other bone metabolic diseases; 3) patients who used glucocorticoids within one year before surgery; 4) vertebral height loss caused by severe kyphosis; 5) severe osteoporosis, bone mineral density (BMD) less than -4.0; 6) infection at sites of surgery; 7) the follow-up time less than 12 months; 8) All the patients who has new compression fractures.

### Assessment of recompression

**Fig 2:**
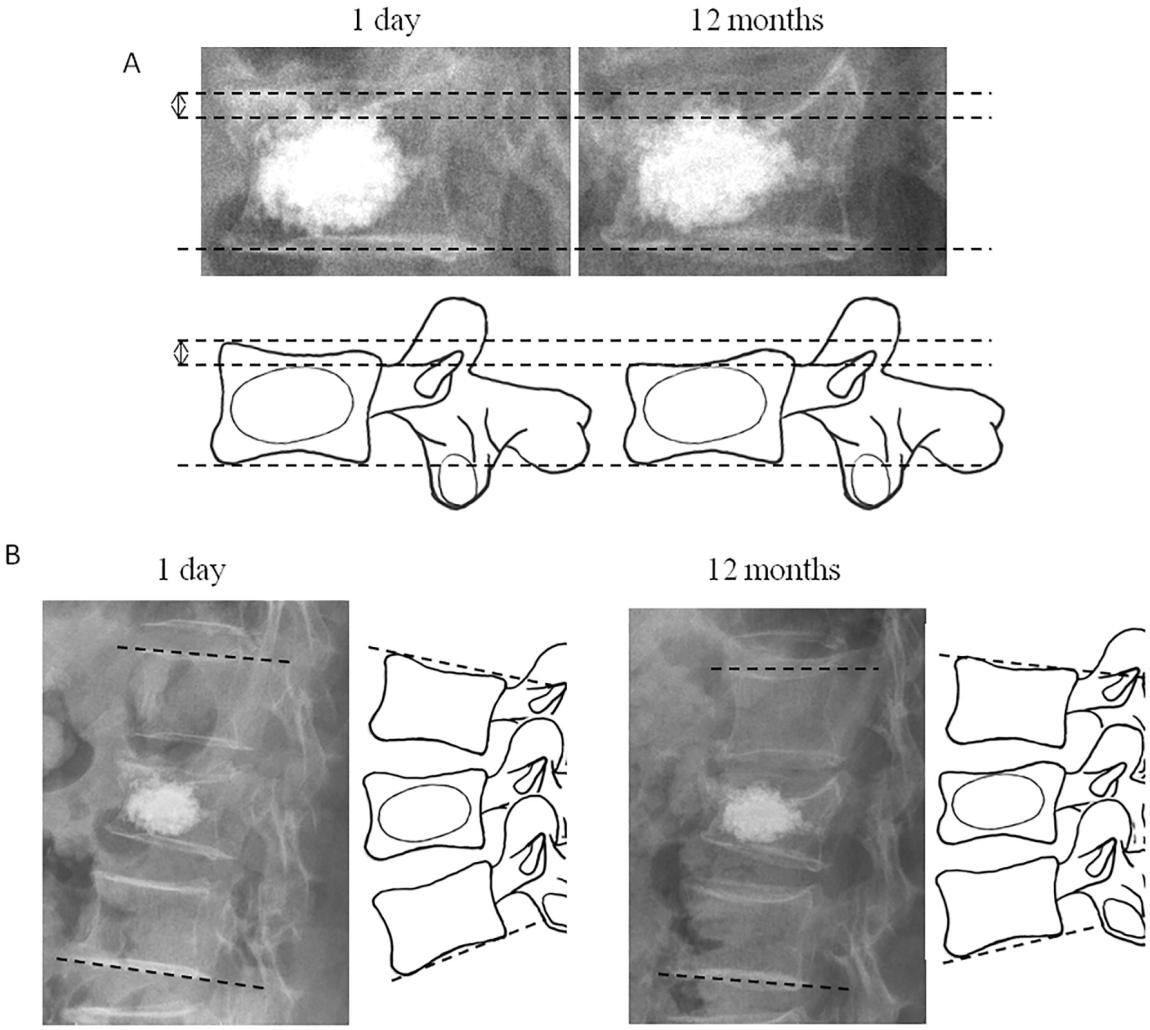
Measurement of vertebral height and kyphosis angle. A. vertebral body height, the distance between the two tangents parallel to the upper and lower endplates lines; B. kyphotic angle was measured using Cobb method: the angle between a line drawn parallel to the superior endplate of the adjacent upper vertebra and the inferior endplate of the lower vertebra.

**Fig 3:**
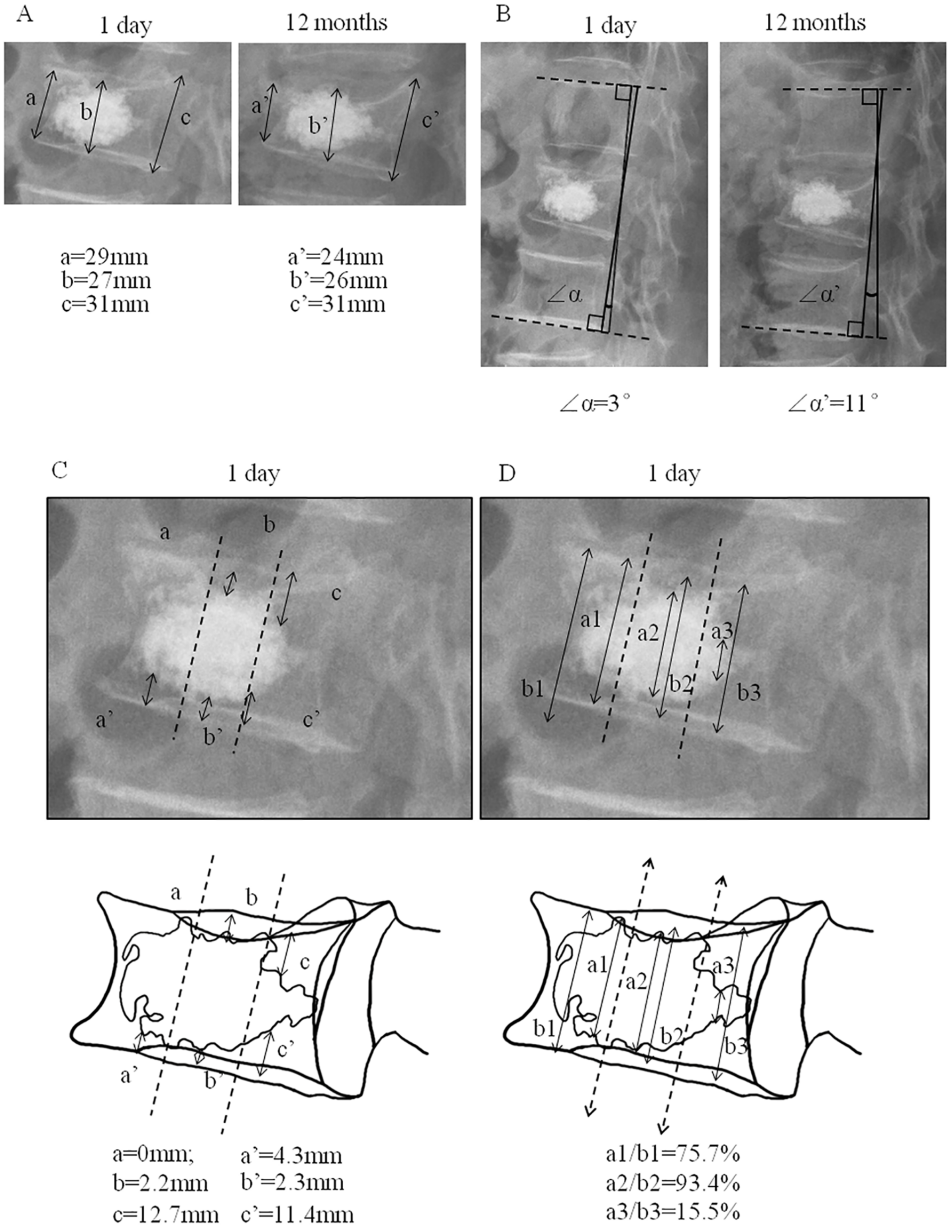
Representative assessment of recompression and imaging analysis. A: X-ray image and the height of different part of vertebral body; B: X-ray image and the kyphotic angle; C: the distance of PMMA to endplates of different part of vertebral body (1 day after surgery); D: the percentage of the height of PMMA in each portion of vertebral body (1 day after surgery); This patient was recorded as “un-contacted” pattern because only the distance of PMMA to upper endplate was zero (anterior part of vertebral body). PMMA = polymethylmethacrylate.

To evaluate the recompression of operated vertebral body, the X-ray films at 1 day after surgery and at 12 months after surgery were compared. The anterior, middle, and posterior heights of the fractured vertebrae were measured on standard supine lateral radiographs using McKiernan’s method[12]. Recompression of the operated vertebral body was defined as following criteria: 1) decreased height of any part of vertebral body > 1.0 mm (Fig 2A); 2) increased kyphosis angle > 3° (Fig 2B), Kyphotic angles were measured using Cobb’s method between the superior endplate of the vertebra directly above and the lower endplate of the vertebra directly below [2]. According to the definition of recompression, all patients were classified into recompression group and non-compression group. Representative assessment of recompression by X-ray images was shown in Fig 3.

### Imaging analysis

**Fig 4:**
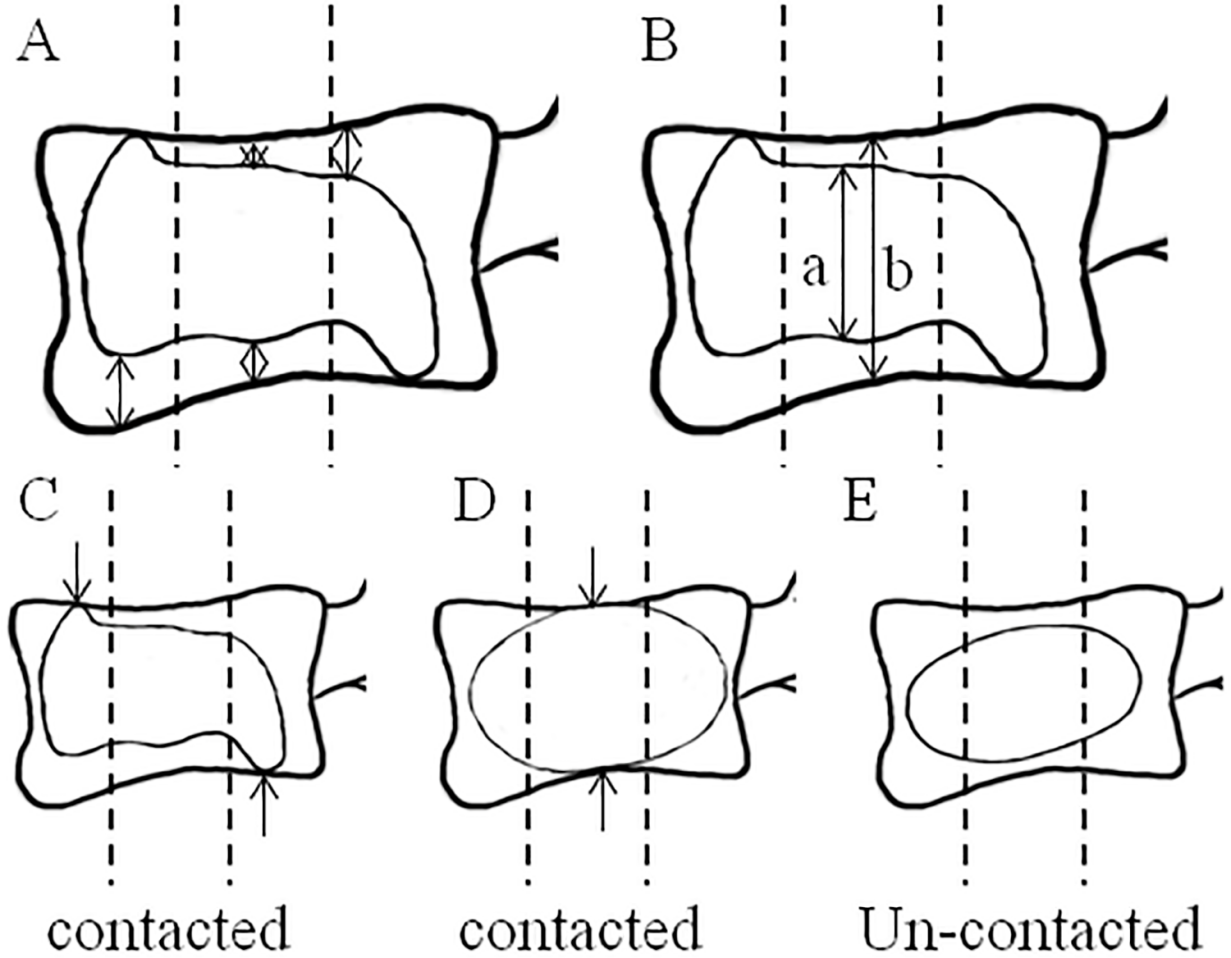
PMMA distribution pattern. A. the minimum distance of PMMA to the endplate; B. percentage of the height PMMA in vertebral body: the highest portion of PMMA as “a”, the highest portion of vertebral body as “b”, the “percentage” was calculated as “a/b”. C, D. the conditions that the minimum distances of PMMA to the upper and the lower endplates were zero at the same time was recorded as “contacted”; E. the other conditions were recorded as “un-contacted”. PMMA = polymethylmethacrylate.

The results of the X-ray films assessment were recorded 1 day after surgery. All films were measured twice individually and independently to eliminate intra- and inter-observer bias by two authors and final decisions were made by consensus. If there was inconsistence, two authors need to re-measure the film together to get the final decision. The imaging data was analyzed using Image-pro plus 5.0 software (Microsoft Corporation, Redmond, WA, USA). The images were measured by the following methods: 1) the minimum distance of PMMA to the upper and lower endplates: the vertebral body was evenly divided into the anterior, middle and posterior portions from the lateral radiograph, the minimum distances of PMMA to the upper and lower endplate of each portion were recorded (Fig 4A); 2) the percentage of the height of PMMA in each portion of vertebral body (the height of the PMMA/the height of vertebral body in each portion [anterior, middle and posterior]) (Fig 4B); 3) contact or un-contacted: the condition that the minimum distances of PMMA to the upper and the lower endplates were zero at the same time was recorded as “contacted” (Fig 4C and 4D), the other conditions were recorded as “un-contacted” (Fig 4E). Representative X-ray images analysis was shown in Fig 3.

### Statistical analysis

SPSS 21.0 software (SPSS Inc., Chicago, IL, USA) was used for statistical analysis. Categorical data between two groups were analyzed using chi-square test. Continuous data between two groups were compared using *t*-test. Multivariate logistic regression analysis was performed, in which the PMMA distribution patterns were used as independent variables and occurrence of recompression was used as dependent variables to evaluate the potential risk factors for recompression.

## Results

One hundred and six (37.7%) patients experienced recompression after surgery during the follow-up period. There was no significant difference in patient demographics. The average age of the patients with recompression was 78.47±7.26 (range, 60–87) and the average age of the patients without recompression was 78.08±8.15 (range, 51–89) (*t* = 0.40, *p =* 0.69). There were 106 patients (36 men and 70 women) in the recompression group and 175 patients (72 men and 103 women) (*χ*^*2*^ = 1.44, *p =* 0.23) in the non-recompression group. The mean BMD T-scores in the recompression and non-recompression groups were -2.73±0.40 and -2.66±0.46 (*t =* -1.24, *p =* 0.22) before surgery and -2.93±0.45 and -2.83±0.43 (*t =* -1.86, *p =* 0.06) after 12 months.

Distributions of PMMA in recompression and non-recompression group were shown in Table 1. The polymethylmethacrylate distributed in the middle of vertebral body showed significant differences between two groups. In non-recompression group, the PMMA in the middle portion of vertebral body were closer to endplates than that in recompression group (upper: *t* = 31.41, *p*<0.001; lower: *t* = 12.19, *p*<0.001). In addition, the percentage of the height of PMMA in the middle portion of vertebral body in non-recompression group is significant higher than that in recompression group **(***t* = -12.92, *p*<0.001**)**. There were 112“contacted” PMMA distribution pattern in non-recompression group and 15 in recompression group, which showed significant difference (*χ*^*2*^ = 66.23, *p*<0.001). Representative MR and X-ray images of recompression and non-recompression were shown in Fig 1.

**Table 1:** The pattern of PMMA distribution.

Minimum distance	Recompression	Non-recompression	*t*	*p* value
**Anterior portion of vertebral body**				
**PMMA to upper endplate**	2.59±0.87	2.46±0.62	1.29	*0*.*20*
**PMMA to lower endplate**	3.02±0.56	3.01±0.69	0.14	*0*.*89*
**percentage of the height**	55.82±11.73%	58.53±11.27%	-1.92	*0*.*056*
**Middle portion of vertebral body**				
**PMMA to upper endplate**	1.99±0.6	0.13±0.17	31.41	*<0*.*001*
**PMMA to lower endplate**	4.00±0.56	3.11±0.72	12.19,	*<0*.*001*
**percentage of the height**	60.96±12.23%	80.07±11.85%	-12.92	*<0*.*001*
**Posterior portion of vertebral body**				
**PMMA to upper endplate**	3.03±0.58	2.99±0.69	0.47	*0*.*64*
**PMMA to lower endplate**	4.00±1.11	3.96±1.15	0.32	*0*.*75*
**percentage of the height**	38.09±11.98%	39.55±11.29%	-1.03	*0*.*31*

Multivariate logistic regression analysis was performed to identify the risk factors associated with the distribution of PMMA. The results were shown in Table 2. The vertebra with higher distance from PMMA to upper or lower endplate in the middle portion of vertebral body has higher risk of recompression after VP or KP (upper endplate: odds ratio [OR] = 6.14, *p*<0.001; lower endplate: OR = 4.03, *p*<0.001). The higher percentage of the height of PMMA in the middle portion of vertebral body indicates the lower risk of recompression (OR<0.01, *p*<0.001). The vertebra with a “contacted” PMMA distribution pattern has lower risk of recompression (OR = 0.09, *p*<0.001).

**Table 2:** Risks of recompression related to PMMA dispersion.

Dispersion of PMMA	*p* value	OR	95%CI	
			Lower	Upper
**Anterior part of vertebral body**				
**PMMA to upper endplate (unit = 1mm)**	0.17	1.27	0.91	1.77
**PMMA to lower endplate (unit = 1mm)**	0.89	1.03	0.71	1.49
**Ratio of the height (unit = 1%)**	0.06	0.13	0.15	1.06
**Middle part of vertebral body**				
**PMMA to upper endplate (unit = 1mm)**	< 0.001	6.14	4.14	9.12
**PMMA to lower endplate (unit = 1mm)**	< 0.001	4.03	2.69	6.03
**Ratio of the height (unit = 1%)**	< 0.001	<0.01	0.00	0.003
**Posterior part of vertebral body**				
**PMMA to upper endplate (unit = 1mm)**	0.64	1.09	0.75	1.58
**PMMA to lower endplate (unit = 1mm)**	0.75	1.04	0.84	1.28
**Ratio of the height (unit = 1%)**	0.30	0.33	0.04	2.71
**Contact**	< 0.001	0.09	0.05	0.17

## Discussion

Some studies have attempted to identify factors related to recompression of previously treated vertebrae after KP or VP [13,14]. In our study, we find the distribution pattern of PMMA in the middle portion of vertebral body was important factor related to recompression. That PMMA in the middle portion of vertebral body was closer to endplates and has higher percentage of the vertebral body height indicates the lower risk of recompression. Furthermore, the vertebra with a “contacted” PMMA distribution pattern has lower risk of recompression.

After VP or KP, although the vertebral body was augmented by PMMA, the range of functional spinal unit motion was not restored to the intact levels [11,15–17]. Due to the effect of motion and pressure, the most weakness part of the vertebra will continue to compress. Biomechanical studies had confirmed that the anterior and posterior region of the lumbar endplates were stronger than the middle region [8–10]. Wilke et al. conducted biomechanical studies using cadaver vertebrae treated with PMMA [18]. They found that subsidence at the center of the upper endplate was greater [18]. Therefore, in order to prevent recompression, PMMA should be well distributed in the middle of the vertebral body.

In our study, we found that smaller distance of PMMA to endplates and higher percentage of the height of vertebral body led to lower incidence of recompression. This effect may be associated with osteoporosis [19]. Osteoporosis with loss of trabeculae always led to continuous loss of vertebral body height and this procedure did not terminate after VP or KP because the osteoporosis is almost impossible to be cure immediately. Kim et al. conducted biomechanical study using osteoporotic cadaveric fractured vertebral bodies to investigate the behavior of fractured osteoporotic vertebral bodies treated with either VP or KP under repetitive loading conditions[11]. In this study, the effect of cyclic loading conditions on treated osteoporotic vertebral fractures was measured after VP or KP and they found the vertebral bodies showed significant height loss during cyclic loading. They found the load should be evenly transmitted through the upper endplate, cancellous bone underneath the upper endplate, filled PMMA, cancellous bone on the lower endplate, and lower endplate, in that order. The non-cemented cancellous bone, whose trabeculae seemed to crush progressively under repetitive loading conditions, is the weakest link in this chain of force transmission[11]. Cyclic loading compressed the osteoporotic cancellous bone between PMMA and endplates and resulted in height loss [11,20]. Therefore, adequate PMMA and higher percentage of PMMA in the height of vertebral body could effectively reduce the loss of vertebral height caused by the compression of cancellous bone.

The relationship of PMMA and endplates is important factor related to recompression. Zhang found that patients with PMMA distributed around both the upper and lower endplates had a lower risk of recompression when compared to patients with bone cement distributed in the middle of vertebral body [4]. Kim found intervertebral cleft and non-PMMA-endplate-contact have close relationship with recompression. Heo et al. did retrospective study and found recompression occurred mainly in the ‘PMMA-non supported area’ [2]. Our finding is consistent to the previous studies. We found that patients with a “contacted” PMMA distribution pattern had lower rate undergoing recompression. The vertebral stiffness of vertebral body was provided by both PMMA and non-cemented bony area after VP or KP, [21,22]. Since the osteoporosis of patients couldn’t be cured immediately, the supporting effect of PMMA was more important. The pressure transferred through the non-cemented bony area would decrease if the PMMA contacted both endplates [11]. Although stress-shielding effect would occur, the collapse of non-cemented bony area caused by pressure would decrease.

## Limitations

The main limitations of this study are its confounders like scoliosis or rotational problems, because only sagittal X-ray images were used to determine the distribution of bone cement. Moreover, although the measurement of vertebral body height and kyphotic angles in the present study has being widely used, the line placement is still relatively subjective. *T*he criterion that decreased anterior body height of >1.0 mm to assess the recompression is so sensitive that false positives is possible to happen. Finally, we found the severity of compression before surgery may also affect the distribution of PMMA and recompression. However, although severely compressed vertebral body before surgery may have lower possibility of recompression, the total loss of vertebral height may increase compared with mildly compressed vertebral body.

## Conclusion

In conclusion, either more PMMA in the middle portion of vertebral body or “contacted” PMMA distribution pattern had a significantly less incidence of recompression. The findings indicated that the control of PMMA distribution during surgery may reduce the risks of recompression after VP or KP.

## Supporting information

S1 STROBE Checklist (DOC)
